# Oncological Outcomes and Quality of Life After Rectal Cancer Surgery

**DOI:** 10.1515/med-2019-0075

**Published:** 2019-09-12

**Authors:** Roberto Peltrini, Gaetano Luglio, Gianluca Cassese, Alfonso Amendola, Emanuele Caruso, Michele Sacco, Gianluca Pagano, Viviana Sollazzo, Antonio Tufano, Mariano Cesare Giglio, Luigi Bucci, Giovanni Domenico De Palma

**Affiliations:** 1 Department of Clinical Medicine and Surgery. University of Naples “Federico II”, 80131 Naples, Via Pansini 5, Italy; 2 Department of Public Health. University of Naples “Federico II”, Naples, Italy; 3 Department of Urology, University of Rome “La Sapienza”, 00161 Roma RM Italy; 4 Center of Excellence for Technical Innovation in Surgery (CEITC). University of Naples Federico II, 80131 Naples, Italy

**Keywords:** Rectal cancer, Laparoscopy, Robotic surgery, Ta-TME, TME, Total mesorectal excision, Quality of life

## Abstract

Surgery for rectal cancer has been completely revolutionized thanks to the adoption of new technologies and up-to-date surgical procedures that have been applied to the traditional milestone represented by Total Mesorectal Excision (TME).

The multimodal and multidisciplinary approach, with new technologies increased the patients’ life expectancies; nevertheless, they have placed the surgeon in front of newer issues, represented by both oncological outcomes and the patients’ need of a less destructive surgery and improved quality of life.

In this review we will go through laparoscopic, robotic and transanal TME surgery, to show how the correct choice of the most appropriate technique, together with a deep knowledge of oncological principles and pelvic anatomy, is crucial to pursue an optimal cancer treatment. Novel technologies might also help to decrease the patients’ fear of surgery and address important issues such as cosmesis and improved preservation of postoperative functionality.

## 1 Introduction

Rectal cancer surgery has been completely revolutionized in recent decades. The Total Mesorectal Excision (TME) concept with neoadjuvant chemo-radiotherapy has significantly improved oncological outcomes, especially in terms of local recurrences [1, 2, 3]. Before TME surgery, local recurrences reached up to 25% [4]. Nowadays their rate is generally lower when TME surgery is associated with radiotherapy [5]. On the other hand, overall survival has not shown the same benefits from the improved disease control as it is primarily influenced by the risk of distant metastasis.

Despite the significant improvements of treatment strategies and oncological outcomes, surgeons are currently challenged by patients’ need for a better quality of life. In fact, regardless of the possibility of recovering from cancer, patients are often frightened by the surgery itself: rectal cancer surgery is generally considered as highly destructive due to the risk of definitive colostomy, incontinence, sexual and urinary dysfunctions, alteration of body anatomy, and overall poor quality of life.

The introduction of new technologies and surgical approaches is the surgeons’ attempt to address these patients’ needs. Today, technology has a strong impact on the management of colorectal malignancy, starting from the availability of new diagnostic tools that allow an early diagnosis. In this regard, a crucial role is played by new endoscopic devices [6]. Future scenarios include the possibility of an “in vivo” histology and an early diagnosis during the endoscopic examination through the so called “augmented endoscopy”. For example, the clinical implementation of confocal laser endomicroscopy (CLE), allows the study of tumours’ vascular models and even to detect early dysplastic lesions [7,8].

Laparoscopy, Robotic, Transanal Total Mesorectal Excision (Ta-TME) and Transanal Minimally Invasive Surgery (TAMIS), are current approaches that perform minimally invasive procedures for rectal cancer surgery. They have been proposed mainly to overcome technical challenges in difficult pelvic dissections, but no evidence exist to date demonstrating that such techniques provide benefits in functional and oncological results, other than improving cosmesis and offering better recovery outcomes.

In this review, we will discuss the current advances of rectal cancer surgery, trying to emphasise the rationale of new techniques and surgical approaches, also on the basis of anatomical and histological considerations, as well as patients’ quality of life related issues.

## 2 Laparoscopic surgery for rectal cancer

Current rectal cancer treatment is based on multimodal management and essentially on chemoradiotherapy and surgery with total mesorectal excision (TME) [9]. TME has become the gold standard for rectal cancer despite the best surgical approach (laparotomy, laparoscopy, robotics, etc.) being still debated, especially for complex cases (obese patients, T4 tumors, very narrow pelvis etc) whereas laparoscopic surgery is sometimes technically demanding. Indeed, data concerning the role of minimally invasive approaches to rectal cancer treatments are sometimes still controversial. On the other hand, the results of several multicentre randomized trials have demonstrated that laparoscopic colon cancer treatment provided benefits in terms of cosmesis, short term outcomes and recovery parameters, with oncological outcomes not inferior to those provided by open surgery [10,11]. Colon cancer laparoscopy has also produced high-quality specimens and is shown to be safe even if performed by surgeons in training [12,13]. The same level of evidence and the benefits of laparoscopic surgery have not been clearly demonstrated in the field of rectal cancer surgery.

Potential benefits of laparoscopy in the surgical treatment of rectal cancer are the same as for colon cancer, namely: shorter hospital stay, reduced intraoperative blood loss, smaller surgical scars, less pain after surgery, faster return to normal activity, and reduced risk of infection without any increase of perioperative mortality.

Data from literature are also difficult to interpret considering that many studies comparing open versus laparoscopic surgery do not include large tumours such as T4 or T3 tumours closer than 2 mm from the endopelvic fascia before neoadjuvant treatments (which often occur in anterior tumors) [14]. On the other hand, some literature reports suggest that T4 colorectal cancer should not always represent an absolute contraindication to laparoscopic approach, making the matter even more confusing [15].

### 2.1 Oncological outcomes after laparoscopic rectal cancer surgery

In 2014, Jeong et al.[16] presented the results of a randomized controlled trial comparing open versus laparoscopic surgery for mid and low rectal cancer requiring neoadjuvant chemo-radiotherapy (COREAN trial). They showed how the oncological results after laparoscopic resection for advanced rectal cancer are not inferior to open surgery. Three hundred and forty patients were enrolled (170 in the laparoscopic group and 170 in the open group) with tumours localized no more than 9 cm away from the anal verge. Three-year disease-free survival was 72.5% after open surgery and 79.2% after laparoscopy, while overall survival was respectively 90.4% and 91.7%. Lastly, local recurrence was 4.9% in the open group and 2.6% in the laparoscopic group.

In 2015, Stevenson and colleagues presented the short term results of the ALaCart study (Effect of Laparoscopic-assisted resection versus open resection on Pathological outcomes in rectal cancer), a randomized non inferiority phase 3 trial [17]. The trial did not demonstrate the non-inferiority of laparoscopic surgery compared to open surgery as far as the pathological quality of the surgical specimens showed. Four hundred and seventy patients were enrolled (238 in the laparoscopic resection group and 237 in the open resection group). The primary endpoint was a composite of oncological factors indicating an adequate surgical resection, in particular the completeness of total mesorectal excision with an intact mesorectal fascia, a clear circumferential resection margin (≥1 mm) and a clear distal margin (≥1 mm). Secondary outcomes were morbidity, mortality, disease-free survival, local pelvic recurrences, quality of life, sexual, bladder and bowel functions. The criteria for high-quality surgery were met for 194 patients in the laparoscopic group (82%) and 208 patients (89%) in the open group; in the laparascopic group, 222 patients (93%) had a clear circumferential resection margin compared to 228 patients (97%) in the open group. Furthermore, in the laparoscopic group 236 patients (99%) had a clear distal margin similarly to the open group ( 234 patients, 99%). Finally, 206 patients (87%) in the laparoscopic group and 216 (92%) in the open group presented a total mesorectal excision with an intact mesorectal fascia.

The two-year follow-up results of the ALaCart trial have been recently published [18]. The authors reported data of the secondary outcomes such as loco-regional recurrence (LRR), disease-free survival (DFS) and overall survival (OS). During a four-year recruitment period between March 2010 and November 2014, 475 patients were enrolled and 450 were included in secondary outcome analyses.

Regarding LRR, the laparoscopic surgery arm showed a 5.4% rate, whereas the open surgery 3.1%. Disease-free and overall survival rates showed slight differences: 80% of laparoscopic patients versus 82% of patients undergoing open surgery were disease-free, while the overall survival rate was 94% versus 93% in the two groups. In conclusion, even if these results are slightly in favour of an open surgical approach, authors concluded that remarkable differences were not demonstrated in 2 years LRR, DFS and OS rates between laparoscopic and laparotomic surgery for rectal cancer.

The ACOSOG Z6051 randomized clinical trial by Fleshman et al. [19] also failed to demonstrate the non-inferiority of laparoscopic approach compared to open surgery for rectal cancer treatment. This multicentric study enrolled 488 patients with stage II and III rectal cancer localised within 12 cm from the anal margin; 240 patients underwent laparoscopic surgery while 222 patients received a laparotomy. A successful resection, meaning as the achievement of high quality specimens as far as the circumferential distal margin and the integrity of the mesorectal fascia, was obtained in 81.7% of patients (95% CI 76.6 - 86.6%) in the laparoscopic group versus the 86.5% of cases (95% CI 82.5-9.4%) in the open group. A negative circumferential radial margin was obtained in 87.9% of patients after laparoscopic surgery and in 92.3% of them after open surgery; again, a complete total mesorectal excision was achieved in a larger number of patients undergoing open surgery (86.9% versus 81.7%). The authors concluded that current data do not support the laparoscopic surgery as a routine clinical practice for rectal cancer treatment even though definitive results from clinical trial are still awaited. Two-years follow-up results from this trial have also been recently published, focusing on disease-free survival and locoregional recurrence [20]. No statistical difference was found between the LAP group and the OPEN one in terms of DFS (79.5% vs 83.2% respectively). Moreover, the laparoscopic technique was not found to increase local recurrences if compared with the laparotomic approach (LAP 2.1%, OPEN 1.8%) and similar results were seen for distant metastases too (LAP 14.6%, OPEN 16.7%).

With regard to long-term results of the above-mentioned studies, it is undeniable that we need to look for larger and well-selected samples, and for longer term observations, to have a clearer idea on the results of laparoscopic rectal surgery, as far as it concerns all the possible outcomes.

Bonjer et al. [21] came to different conclusions due to the results from the COLOR II trial (A randomized Trial of Laparoscopic versus Open surgery for rectal cancer), a non-inferiority, open label, multi-centre trial. They recognized that laparoscopic surgery in patients with rectal cancer is safe and effective with both local recurrence and overall and disease-free survival rates similar to those achieved through the open surgery approach. One thousand and forty-four patients were enrolled in the trial (345 in the open group and 699 in the laparoscopic group), although patients with T4 cancer and T3 lesions within 2 mm from the endopelvic fascia were excluded being at higher risk for local recurrences. The primary endpoint was the loco regional recurrence rate which resulted as follows: upper rectal tumours: 3.5% for the laparoscopic group and 2.9% for the open group; middle rectal tumours: 6.5% and 2.4% respectively; lower rectal cancer: 4.4% in lap and 11.7% in the open group. The overall recurrence rate was 5% in both groups. Three-year disease-free survival rate was 74.8% in the laparoscopic group and 70.8% in the open group, while overall survival rate was 86.7% after laparoscopy and 83.6% after open surgery. Finally, distant metastases were identified in 19.1% of patients in the laparoscopic group and 22.1% in the open group at three year follow-up. These data demonstrate not only that laparoscopic surgery in rectal cancer is oncologically safe but also the oncological results seem to be comparable to those achieved after open surgery.

It is also important to discuss additional issues concerning laparoscopic surgery of the rectum. In fact, rectal cancer laparoscopy is commonly considered as technically difficult. Then, the risk of conversion has to be taken into account when considering short-term outcomes, even if it does not significantly compromise morbidity (with the exception of a higher rate of wound infections) or oncological outcomes [22,23]. The conversion rate widely varies between trials: it is reported between 1.2% (COREAN trial) and 34% (CLASSIC trial). The main reasons for conversion are obesity, narrow pelvic anatomy, uncontrollable bleeding, ureteral injury and advanced disease.

The data from AlaCart and ACOSOG Z6051, compares the laparotomic operative time with the laparoscopic approach for rectal cancer and shows that laparoscopy usually has a longer operative time (210 minutes versus 190 minutes in AlaCart and 266 versus 220 minutes in ACOSOG Z6051). Similar results have been reached by a recent meta-analysis of 14 studies (7 randomized and 7 non-randomized) reporting longer operative time after laparoscopy [24].

With regard to the length of hospital stay, studies report similar results from the two groups. In fact, the ACOSOG Z6051 reports a length of in-hospital stay of 7.3 days in the laparoscopic group and of 7.0 days in the open group while ALaCart trials show a length of 8 days for both groups. The above mentioned meta-analysis also reports the results from seven studies evaluating the recovery time for the intestinal function which is actually shorter after laparoscopy [24].

Most of literature data are controversial about laparoscopic rectal cancer surgery, maybe because of the huge amount of data in literature. It should be pointed out that the most of these results are mainly based on postoperative histological assessment rather than clinical endpoints, making the interpretation of such results difficult.

Based on current evidence we should probably conclude that despite its appeal, laparoscopic TME is beneficial in selected cases but data from literature also emphasise some drawbacks, particularly the technical challenges that make rectal dissection difficult, sometimes compromising the specimen quality. Therefore, the surgeon’s expertise in laparoscopic surgery is a key factor with a major impact on oncological and perioperative outcomes.

## 3 Robotic surgery for rectal cancer

Rectal cancer laparoscopy has not achieved the success it deserves despite the remarkable development of minimally invasive surgery in recent decades. This is probably due to the technical complexity of TME, the very steep learning curve for young surgeons, and of the complex pelvic anatomy. Robotic surgery has the appeal of overcoming these fundamental limits thanks to its three-dimensional vision together with better ergonomics and superior precision of TME due to the robotic arms technology. Total mesorectal excision with transection of the rectum at the anal canal is usually required for middle and lower rectal cancers. With regard to the upper rectum tumours, PME (partial mesorectal excision) is usually adequate even though you have to preserve an intact mesorectal fascia, ensuring a 5-cm distal mesorectal resection margin [25,26]. On the other hand, coloanal anastomosis with intersphinteric resection is a reasonable alternative for very low tumours if they do not invade the pelvic floor muscles. It preserves sphincter function, thus avoiding abdominoperineal resection (APR), and allows a good quality of life; these techniques have been described in minimally invasive as well as in open procedures [27,28].

Two main approaches are usually adopted for robotic assisted TME: the hybrid technique and the full robotic.

The hybrid technique consists of laparoscopic mobilization of the left colon plus splenic flexure mobilization followed by robotic total mesorectal excision. The totally robotic resection is carried out with the aid of the four-arm Da Vinci’s robotic system. The patient is placed in lithotomy position while the robot is docked at the patient’s left side. The operation begins with the medial to lateral mobilization of the left colon, with the high ligation of the mesenteric vessels. Once the left colon and the splenic flexure are completely mobilized, the pelvic TME dissection begins. Rectal transection is usually performed laparoscopically with a linear stapler. The specimen is taken out through a supra-pubic mini-laparotomic access; in the end, a laparoscopic coloanal anastomosis is then perfomed [29].

The first study on robotic TME was led by Pigazzi et al. [30] in 2006. In this study the authors compared the laparoscopic approach with the new robotic technique. In particular, they compared the results in 6 patients undergoing laparoscopic rectal resection with six patients operated using the robot. Regarding short-term outcomes, the operative time was similar for the two groups (4.3 hours versus 4.4 hours), while the operative blood loss was higher in the laparoscopic group (150 cc vs 104 cc); shorter in-hospital stay was reported for patients undergoing laparoscopy (3.6 days vs. 4.5 days). After this preliminary experience, the authors deduced that robotic surgery has the ability to get less demanding rectal dissections that is especially beneficial to young surgeons trained in minimally invasive colon surgery.

Overall, literature data dealing with safety, feasibility and outcomes after robotic rectal surgery are often controversial and a definitive answer regarding the best approach for surgical treatment of rectal cancer probably does not exist to date.

### 3.1 Oncological outcomes after robotic rectal cancer surgery

in 2010, Pigazzi et al. [29] published results from a multi-center study including patients undergoing robotic rectal resection in three referral centres from November 2004 to December 2008. One hundred and forty-three patients were recruited, 112 received an anterior resection and 31 an abdominoperineal amputation. Oncological outcomes were then evaluated: pathologic complete response to radiotherapy was observed in 18 patients (accounting for 19.3% of irradiated patients); distal and circumferential resection margins were negative in 142 out of 143 cases; 3-year disease-free survival was 77.6% and 3-year overall survival was 97%, while 11 patients (7.7%) developed distant metastases. In addition, 3 deaths were recorded (1 for non-interventional pneumonia and 2 for distant metastases); no port-site or local recurrences were recorded.

In 2012 Yang et al. published a meta-analysis including 16 studies from January 2000 to July 2011 [31]. They compared conventional laparoscopic surgery with robotic surgery, including 1493 patients from 6 centres (1125 with malignant diseases and 368 with benign ones). The authors did not demonstrate significant differences in the number of lymph nodes retrieved after both approaches. The same conclusion was also found for distal margins and circumferential resection margins (CRM).

In 2015, Park et al. analyzed outcomes from 217 patients with rectal cancer [32]. They were divided into robotic and laparoscopic arms. In particular, 133 patients underwent robotic surgery and 84 laparoscopic surgery (the remaining patients dropped out because they either received open surgery or presented an advanced stage 4 disease). The oncological outcomes were similar in terms of number of lymph nodes retrieved (16.34 +/ - 8.79 for the robotic group and 16.63 +/- 10.24 for the laparoscopic group), as well as the CRM, that was positive in 6.8% of patients in the robotic group and in 7.1% of patients in the laparoscopic group. Five year overall survival was 92.8% in patients operated on with the robot and 93.5% in those operated on laparoscopically; finally, 5-year disease free survival was 81.9% in the robotic group and 78.7% in the laparoscopic group. Despite similar oncological outcomes, authors were not able to show benefits of the robotic surgery compared to laparoscopy, particularly in the light of higher cost required for robotic procedures.

In 2017 Jayne et al. reported outcomes from a randomized clinical trial conducted at 29 sites, including 471 patients with rectal adenocarcinoma operated on from January 2011 to September 2014 (237 patients were randomized to receive robotic assisted surgery vs 237 patients operated on laparoscopically) [33]. Most tumours were staged as pT2 and pT3. The number of lymph nodes retrieved was adequate and similar in both the laparoscopic and the robotic arm. No significant differences were found in terms of CRM involvement; this was positive in 26 patients (5.7%), 14 in the robotic arm and 12 in the laparoscopic one. Furthermore, no proximal margin involvement was observed in any patient, whereas a single patient had a distal margin involved. The quality of TME was similar in both groups.

Postoperative morbidity after robotic surgery includes the well known complications typical of colorectal surgery: possible bleeding, iatrogenic injuries to pelvic structures, bowel obstruction, wound infection, anastomotic leak and postoperative urinary and sexual dysfunction. Nevertheless, complication rates from most studies are similar to those reported for laparoscopic surgery.

The most common cause of conversion is usually the inability to perform a correct pelvic dissection, mainly because of obesity associated with heavy mesorectum or narrow pelvis.

Pigazzi et al. reported a laparotomy conversion rate of 4.9% (7 patients out of 143), six of them were male with complex and narrow pelvic anatomy [29]. Jayne et al. led out the ROLLAR randomized trial to evaluate the risk of conversion in robotic vs laparoscopic rectal resection, in order to assess if robotic could overcome technical difficulties of conventional laparoscopy in challenging cases: laparotomic conversion rate in robotic group was 8.1% vs 12.2% in the laparoscopic group [33]. As predictable, the conversion rate was higher in men than in women; in fact, the laparotomic conversion from robotic operations was necessary in 14 out of 161 males (8.7%) and in 5 out of 75 females (6.7% ); with regard to the laparoscopic group, the conversion to open surgery was necessary in 25 out of 156 males (16.0%) and in 3 out of 74 (4.1%). The different rates reported in the two groups were not significant and authors from ROLARR concluded that robotic does not confer an advantage for rectal cancer resection so far.

On the other hand, most studies demonstrate that robotic rectal surgery is associated with longer operative time when compared with traditional laparoscopy. Specifically, results from ROLARR Trial show that robotic rectal resection requires an average of 37.5 minutes longer.

Results from literature are controversial and a great heterogeneity exists among studies; for example, a meta-analysis of eight studies [34] shows how robotic is associated with a lower rate of positive circumferential resection margins and erectile dysfunctions, other than a lower conversion rate; whereas, operative time, morbidity and recovery parameters do not significantly differ from those reported for laparoscopic procedures.

The data heterogeneity evidenced by studies and meta-analysis makes their interpretation very difficult; more focused trials scheduling longer follow-up are needed to definitively assess the robotic role in rectal cancer management. In this regard it is worth pointing out an important phase II open label prospective randomized controlled trial by Kim et al. that is currently underway [35], in order to compare the quality of TME specimen (the primary outcome), CRMs, DRM, the number of harvested lymph nodes, morbidity, bowel function recovery, and quality of life (all secondary outcomes). A total of 163 patients staged cT1-3NxM0 were randomly assigned to the robot-assisted (n = 81) and laparoscopic (n = 82) surgery groups, and 139 patients were eligible for the analyses (73 vs 66, respectively). Only one patient was converted to open surgery from the robotic group. The TME quality was found to be similar in the robot-assisted and laparoscopic groups (80.3% vs 78.1% complete TME, respectively). The resection margins, number of harvested lymph nodes, morbidity, and bowel function recovery also were not significantly different. With regard to quality of life, the two techniques also showed similar results, as further discussed.

## 4 Transanal total mesorectal exicision (TaTME)

Trans-anal Total Mesorectal Excision (Ta-TME) was firstly introduced by Lacy et al. in 2010 [36,37]. It is based on the combination of minimally invasive abdominal and perineal approaches to complete the distal rectal dissection in order to overcome those technical issues which are typical of both laparoscopic and robotic rectal surgery.

As stated by the *St. Gallen Colorectal Consensus Expert Group*, TaTME would be preferably indicated in cases of mid-distal bulky tumours, obese patients and narrow pelvis. It has the potential benefit of an easier intersphincteric resection - when indicated - and a better nerve preservation thanks to an appropriate view of distal rectum, especially its intra-elevator part, which is known as the “no man’s land”. Nevertheless, this procedure has some drawbacks as emphasized by preliminary reports: dissection in the proper anterior plane might be tricky, with the risk for urethral, vaginal or prostatic injuries [38]; moreover, an appropriate learning curve is obviously required [39], because of the loss of the usual anatomic landmarks, as the distal resection starts from below.

Data from selected series and their meta-analysis encourage this novel approach, even in terms of short-term oncological “surrogate” outcomes. A 2016 meta-analysis reports a better circumferential margin after TaTME with a lower CRM positivity rate and an overall higher TME quality according to Quircke’s criteria [40]. A more recent meta-analysis by Jian et al. concludes that TaTME would be able to achieve a longer distal resection margin (DRM) when it is compared to laparoscopy [41]. Such result might be explained by the advantages of pneumo-dissection, in that the right anatomic planes can be found by better visualization of the distal margin of the tumour (from the luminal side of the rectum before the dissection begins). The CRM positivity rate significantly decreased also in the Bordeaux’ prospective randomized trial which enrolled 100 patients even though, perhaps surprisingly, the 5-year local recurrence rate (2,6% in the TaTME group vs 4,8% in the Lap group) and the 5-year OS were not significantly affected by the surgical approach [42]. Post-operative major morbidity is also comparable with laparoscopic surgery by considering similar rate of postoperative ileus, anastomotic leak and urinary dysfunction.

More robust evidences from multicentre RCTs are certainly advocated to definitively establish the role of TaTME in clinical practice. Hopefully, the long term results of the COLOR III trial [43] and the ETAP-GRECCAR 11 TRIAL [44] will provide these required answers.

## 5 Quality of life: what’s the role of novel technologies?

Quality of life is a priority in modern rectal cancer surgery especially the preservation of postoperative function [45].

Preservation of sphincter function after an ultra-low colorectal, coloanal, inter-sphincteric or even a pull-trough procedure has certainly decreased the patients’ fear for surgery and postoperative impact of surgery itself on social life [27,28,46,47].

The construction of a temporary ileostomy is generally advocated by most authors in case of ultra-low resection or coloanal anastomosis and if its early reversal (usually 8 weeks after primary surgery) is safe. It has a reasonable rate of complications, and is well tolerated by most patients [48,49].

One more frontier of current rectal cancer surgery is the attempt to preserve postoperative urogenital function, namely the adoption of “nerve-sparing” procedures [50]. Nevertheless, the complete preservation of autonomic nerve structures may be unsuitable due to the necessity of an appropriate tumour clearance; thus, a modern-state-of-the-art surgery should aim to pursue the trade off between oncological clearance and function preservation.

Sometimes, patients and tumours characteristics may prevent the possibility to achieve both results, especially in cases of anterior bulky tumours close to or infiltrating the Denonvillier’s fascia, or lateral cancers infiltrating the rectal stalks or pelvic sidewall structures; the same is true for very narrow pelvis especially in obese patients, for previous pelvic surgery and occasionally for radiotherapy.

The identification of the inferior hypogastric plexus and the preservation of hypogastric nerves is considered as the first step to perform good-quality nerve-sparing surgery [51]. On the other hand, emotional, psychological and social elements may also contribute to postoperative sexual and urinary dysfunctions, even if injury of the neurovascular bundle is usually supposed to be the main responsible factor for urinary dysfunction (0-15%) and sexual impairments (10-35%) of patients undergoing TME surgery; moreover, a perfect postoperative function cannot be ensured for all patients even with a meticulous nerve preserving technique [52].

In this context, laparoscopic and robotic surgery have been advocated as potential tools to help in obtaining better nerve preservation and functional outcomes, although results from the literature seem to be quite controversial in this regard.

A subset analysis from the COLOR II trial, for example, comparing open versus laparoscopy, shows no differences in sexual dysfunction and micturition problems [53]. The same seems to be true for robotic; the high magnification and the technical benefits of robotic-assisted approach does not seem to offer better sexual and urinary outcomes when compared to conventional laparoscopic surgery: results from the ROLARR trial do not show statistically significant differences in term of postoperative functional scores (I-PSS, IIEF and FSFI).

On the other hand, Kim et al. led a prospective case-matched study to assess QoL after robotic and laparoscopic surgery [54]. The International Prostatic Symptom Score (I-PSS) was significantly better in the robotic arm at six months; moreover, the I-PSS was comparable to baseline at 6 months in the robotic group and at 12-months in the laparoscopic group. The same was true for the International Index of Erectile Function 5 (IIEF-5) that returned to preoperative level at 6-months in the robotic group and at 12-month in the laparoscopic one

Similar results have been obtained from a meta-analysis by Lee et al. whereas, comparing the I-PSS after robotic and laparoscopic surgery at 3-6 and 12 months, robotic surgery achieved better results at 3 months, although this difference was not maintained by 6 and 12 months [55]. Robotic surgery also scored better in regard to erectile function at 3 and 6 months after surgery.

Finally, Ta-TME needs to be mentioned also for its potential to improve the postoperative functional outcomes; a randomized trial by Pontallier et al. failed to show significant differences in terms of bowel and urinary function between TaTME and conventional laparoscopy, despite a “trend” towards a better erectile function and preserved sexual activity in the transanal group [56].

Kneist et al. showed 10% and 40% of patients having major and minor LARS, respectively, and median LARS score 26 at 6 months after TaTME [57], in one of several studies focusing on intraoperative electrophysiological assessment of hypogastric nerves lesions; on other hand, the median Wexner Score (WS) was significantly higher at month 6 (WS of 7 [moderate incontinence]) after stoma closure than pre-operatively. Outcomes concerning sexual impairment and urinary dysfunction in this study are complex to be objectively interpreted because of the heterogeneity and the small size of the cohort.

Veltcamp Helbach et al. compared Laparoscopic (LAR) and Transanal (TaTME) TME, administered both EORTC QLQ-C30 and C29 questionnaires about QoL, with results in favour of LAR as far as for fatigue, role dysfunction, financial impact, hair loss and faecal incontinence [39]. Items exploring anorectal function in terms of diarrhoea and constipation did not show significant differences, as seen by a LARS score of 24 and 27.7 respectively in LAR and TaTME arm, even considering incontinence to flatus and liquid stool questions. Postoperative IPSS score, female dyspareunia and male impotence were not influenced by surgical approach. When EQ-5D-3L and EQ-VAS was used to analyse overall QoL after TaTME in a prospective cohort study, 1 month outcomes worsened significantly, mostly considering social life impairment, while results show a return to preoperative baseline 6 months after the intervention [58]. This trend was confirmed in EORTC QLQ-C30 questionnaires, excepting for anal pain and social functioning which remained significantly altered at 6 months after procedure. Bladder function, female and male sexual outcomes at 6 months returned to the baseline. Anorectal function analysis showed no significant differences after TaTME, with a major LARS rate of 33.3%, minor LARS in 20%, and no LARS in 46.7% of patients.

Larger multicenter studies are certainly required to obtain definitive answers regarding the role of these novel procedures and their impact on quality of life, possibly overcoming concerns about interpretation of different tests administered, bias related to an adequate learning-curve for TaTME approach, and poor data when female sexual and functional outcomes are analysed.

Potential benefits from novel approaches in rectal cancer surgery might be also analysed from a different prospective: the adequate selection of patients and correct indications for each type of procedure. With this regard, a fundamental role is played by the High-Resolution MRI. Specialists can obtain now detailed information about tumor height, local and venous infiltration, lymph node status. MRI-predicted CRM status presents the same association with Local Recurrences (LR), Disease Free Survival, Overall Survival, than CRM pathologically assessed: we can assume that LR is 20% and 27% in case of MRI-CRM and pathological-CRM positivity respectively, which rises to 32% if both positive and drops to about 7% if both negative [59]. These considerations emphasize the key role played multidisciplinary teams, with the most appropriate choice of the best treatment strategy. From this standpoint a fundamental role is certainly played by preoperative MRI staging, which is able to assess the intersphincteric plane involvement, and enable selection of patients with chance for sphincter-saving surgery. The benefits of optimising medical and surgical management of rectal cancer has been demonstrated by MERCURY II study group [60], showing a reduction to 15% in pCRM involvement, compared to 30% of positivity rate described for low rectal cancer in previous reports.

In addition, MRI pelvimetric parameters correlate with pathologic outcomes influencing short term oncological outcome. Interestingly interspinous distance, and obstetric conjugates in some analysis, seem to predict quality of TME influencing CRM positivity, irrespective from open or laparoscopic assisted approach [61].

## 6 Conclusions

The patients’ need for a good or at least acceptable quality of life is one of the leading aspect of current rectal cancer surgery. Modern technologies, new surgical procedures, together with a deep knowledge of pelvic anatomy and oncological principles, may help the contemporary colorectal surgeon pursue the proper cancer treatment, without giving up the possibility to preserve both cosmesis and satisfactory postoperative functions. The key could be tailored surgery, where the best technique is chosen on a case by case basis and the experience of the surgeon.

## References

[1] Heald R. J., Moran B. J., Ryall R. D., Sexton R., MacFarlane J. K. (1978). Rectal cancer: the Basingstoke experience of total mesorectal excision. Arch. Surg.

[2] Kapiteijn E., Marijnen C.A., Nagtegaal I.D. (2001). Preoperative Radiotherapy Combined with Total Mesorectal Excision for Resectable Rectal Cancer. N. Engl. J. Med.

[3] Van Gijn W., Marijnen C.A., Nagtegaal I.D. (2011). Preoperative radiotherapy combined with total mesorectal excision for resectable rectal cancer: 12-year follow-up of the multicentre. randomised controlled TME trial, Lancet Oncol.

[4] Pilipshen S. J., Heilweil M., Quan S. H.Q., Sternberg S. S., Enker W. E. (1984). Patterns of pelvic recurrence following definitive resections of rectal cancer. Cancer.

[5] Ngan S.Y., Burmeister B., Fisher R.J. (2012). Randomized Trial of Short-Course Radiotherapy Versus Long-Course Chemoradiation Comparing Rates of Local Recurrence in Patients With T3 Rectal Cancer: Trans-Tasman Radiation Oncology Group Trial 01.04. J Clin Oncol.

[6] De Palma G.D., Giglio M.C., Bruzzese D. (2018). Cap cuff-assisted colonoscopy versus standard colonoscopy for adenoma detection: a randomized back-to-back study Gastrointest. Endosc.

[7] De Palma G.D., Maione F., Esposito D. (2016). In vivo assessment of tumour angiogenesis in colorectal cancer: the role of confocal laser endomicroscopy. Colorectal Dis.

[8] De Palma G.D., Colavita I., Zambrano G. (2017). Detection of colonic dysplasia in patients with ulcerative colitis using a targeted fluorescent peptide and confocal laser endomicroscopy: A pilot study. PLoS One.

[9] Benson A.B., Veenook A.P., Al-Hawary M.M. (2018). Rectal Cancer. Version 2.2018, NCCN Clinical Practice Guidelines in Oncology.

[10] Luglio G., Nelson H (2010). Laparoscopy for colon cancer: state of the art, Surg. Oncol. Clin. N. Am.

[11] Fleshman J., Sargent D.J., Green E. (2007). Laparoscopic colectomy for cancer is not inferior to open surgery based on 5-year data from the COST Study Group trial. Ann. Surg.

[12] West N. P., Kennedy R.H., Magro T. (2014). Morphometric analysis and lymph node yield in laparoscopic complete mesocolic excision performed by supervised trainees. Br. J. Surg.

[13] Luglio G., De Palma G.D., Tarquini R. (2015). Laparoscopic colorectal surgery in learning curve: Role of implementation of a standardized technique and recovery protocol. A cohort study. Ann. Med. Surg.

[14] Bonjer H. J., Deijen C. L., Abis G. A. (2015). A Randomized Trial of Laparoscopic versus Open Surgery for Rectal Cancer. N. Engl. J. Med.

[15] Bretagnol F., Dedieu A., Zappa M., Guedj N., Ferron M., Panis Y. (2011). T4 colorectal cancer: is laparoscopic resection contraindicated?. Colorectal Dis.

[16] Jeong S.Y., Park J.W., Nam B.H. (2014). Open versus laparoscopic surgery for mid-rectal or low-rectal cancer after neoadjuvant chemoradiotherapy (COREAN trial): survival outcomes of an open-label. non-inferiority, randomised controlled trial , Lancet. Oncol.

[17] Stevenson A. R., Solomon M.J., Lumley J.W. (2015). Effect of Laparoscopic-Assisted Resection vs Open Resection on Pathological Outcomes in Rectal Cancer. JAMA.

[18] Stevenson A. R. L., Solomon M.J., Brown C.S.B. (2018). Disease-free Survival and Local Recurrence After Laparoscopic-assisted Resection or Open Resection for Rectal Cancer. Ann. Surg.

[19] Fleshman J., Branda M., Sargent D.J. (2015). Effect of Laparoscopic-Assisted Resection vs Open Resection of Stage II or III Rectal Cancer on Pathologic Outcomes: The ACOSOG Z6051 Randomized Clinical Trial. JAMA.

[20] Fleshman J., Branda M., Sargent D.J. (2019). Disease-free Survival and Local Recurrence for Laparoscopic Resection Compared With Open Resection of Stage II to III Rectal Cancer. Ann. Surg.

[21] Bonjer H. J., Deijen C. L., Haglind E. (2015). A Randomized Trial of Laparoscopic versus Open Surgery for Rectal Cancer. N. Engl. J. Med.

[22] Giglio M.C., Celentano V., Tarquini R., Luglio G., De Palma G.D., Bucci L. (2015). Conversion during laparoscopic colorectal resections: a complication or a drawback? A systematic review and meta-analysis of short-term outcomes. Int. J. Colorectal Dis.

[23] Giglio M.C., Luglio G., Sollazzo V. (2017). Cancer recurrence following conversion during laparoscopic colorectal resections: a meta-analysis. Aging Clin. Exp. Res.

[24] Chen K., Cao G., Chen B. (2107). Laparoscopic versus open surgery for rectal cancer: A meta-analysis of classic randomized controlled trials and high-quality Nonrandomized Studies in the last 5 years. Int. J. Surg.

[25] Heald R. J., Husband E. M., Ryall R. D. H. (1982). The mesorectum in rectal cancer surgery - the clue to pelvic recurrence ?. Br. J. Surg.

[26] Leong A. F. P. K. (2000). Selective total mesorectal excision for rectal cancer. Dis. Colon Rectum.

[27] Giglio M.C., Persico M., Quarto G. (2013). Intersphinteric resection for rectal cancer: Role in fecal continence and quality of life. Ann. Ital. Chir.

[28] Luglio G., Masone S., Quarto G. (2013). Functional results after TME: J-pouch vs straight coloanal anastomosis and role of neoadjuvant radiochemotherapy. Ann. Ital. Chir.

[29] Pigazzi A., Luca F., Patriti A. (2010). Multicentric study on robotic tumor-specific mesorectal excision for the treatment of rectal cancer. Ann. Surg. Oncol.

[30] Pigazzi A., Ellenhorn J. D. I., Ballantyne G. H., Paz I. B. (2006). Robotic-assisted laparoscopic low anterior resection with total mesorectal excision for rectal cancer. Surg. Endosc.

[31] Yang Y., Wang F., Zhang P., Shi C., Zou Y., Qin H., Ma Y (2012). Robot-Assisted Versus Conventional Laparoscopic Surgery for Colorectal Disease. Focusing on Rectal Cancer: A Meta-analysis, Ann. Surg. Oncol.

[32] Jung Park E., Cho M.S., Baek S.J. (2015). Long-term Oncologic Outcomes of Robotic Low Anterior Resection for Rectal Cancer A Comparative Study With Laparoscopic Surgery. Ann Surg.

[33] Jayne D., Pigazzi A., Marshall H. (2017). Effect of Robotic-Assisted vs Conventional Laparoscopic Surgery on Risk of Conversion to Open Laparotomy Among Patients Undergoing Resection for Rectal Cancer. JAMA.

[34] Xiong B., Ma L., Huang W., Zhao Q., Cheng Y., Liu J. (2019). Robotic versus laparoscopic total mesorectal excision for rectal cancer: a meta-analysis of eight studies. J. Gastrointest. Surg.

[35] Kim M.J., Park S.C., Park J.W. (2018). Robot-assisted Versus Laparoscopic Surgery for Rectal Cancer: A Phase II Open Label Prospective Randomized Controlled Trial. Ann Surg.

[36] Sylla P., Rattner D. W., Delgado S., Lacy A. M. (2010). NOTES transanal rectal cancer resection using transanal endoscopic microsurgery and laparoscopic assistance Surg. Endosc.

[37] Lacy A.M., Tasende M.M, Delgado S. (2015). Transanal Total Mesorectal Excision for Rectal Cancer: Outcomes after 140 Patients. J. Am. Coll. Surg.

[38] Adamina M., Buchs N. C., Penna M., Hompes R. (2018). St.Gallen Colorectal Consensus Expert Group. St.Gallen consensus on safe implementation of transanal total mesorectal excision. Surg. Endosc.

[39] Veltcamp Helbach M., Koedam T.W.A., Knol J.J. (2019). Quality of life after rectal cancer surgery: differences between laparoscopic and transanal total mesorectal excision. Surg. Endosc.

[40] Ma B., Gao P., Song Y. (2016). Transanal total mesorectal excision (taTME) for rectal cancer: a systematic review and meta-analysis of oncological and perioperative outcomes compared with laparoscopic total mesorectal excision. BMC Cancer.

[41] Jiang H.P, Li Y.S., Wang B. (2018). Pathological outcomes of transanal versus laparoscopic total mesorectal excision for rectal cancer: a systematic review with meta-analysis. Surg. Endosc.

[42] Denost Q., Loughlin P., Chevalier R., Celerier B., Didailler R., Rullier E. (2018). Transanal versus abdominal low rectal dissection for rectal cancer: long-term results of the Bordeaux’ randomized trial. Surg. Endosc.

[43] Deijen C.L., Velthuis S., Tsai A. (2016). COLOR III: a multicentre randomised clinical trial comparing transanal TME versus laparoscopic TME for mid and low rectal cancer. Surg. Endosc.

[44] Lelong B., de Chaisemartin C., Meillat H. (2017). A multicentre randomised controlled trial to evaluate the efficacy. morbidity and functional outcome of endoscopic transanal proctectomy versus laparoscopic proctectomy for low-lying rectal cancer (ETAP-GRECCAR 11 TRIAL): rationale and design, BMC Cancer.

[45] De Palma G. D., Luglio G. (2015). Quality of life in rectal cancer surgery: What do the patient ask?. World J. Gastrointest. Surg.

[46] Rullier E., Laurent C., Bretagnol F., Rullier A., Vendrely V., Zerbib F. (2005). Sphincter-saving resection for all rectal carcinomas: The end of the 2-cm distal rule. Ann. Surg.

[47] Bianco F., Falato A., Belli A., De Franciscis S., De Leon Valdez J.D., Romano G.M. (2017). Modified Pull-Through Technique With a Delayed High Coloanal Anastomosis: no stoma and scarless surgery for low rectal cancer , Dis. Colon Rectum.

[48] Luglio G., Pendlimari R., Holubar S.D., Cima R.R., Nelson H. (2011). Loop ileostomy reversal after colon and rectal surgery: A single institutional 5-year experience in 944 patients. Arch Surg.

[49] Luglio G., Terracciano F., Giglio M.C. (2017). Ileostomy reversal with handsewn techniques. Short-term outcomes in a teaching hospital. Int. J. Colorectal Dis.

[50] Mancini R., Cosimelli M., Filippini A. (2000). Nerve-sparing surgery in rectal cancer: Feasibility and functional results. J. Exp. Clin. Cancer Res.

[51] Kneist W., Kauff D. W., Juhre V., Hoffmann K. P., Lang H. (2013). Is intraoperative neuromonitoring associated with better functional outcome in patients undergoing open TME?. Eur. J. Surg. Oncol.

[52] Celentano V., Fabbrocile G., Luglio G., Antonelli G., Tarquini R., Bucci L. (2010). Prospective study of sexual dysfunction in men with rectal cancer: feasibility and results of nerve sparing surgery. Int. J. Colorectal Dis.

[53] Andersson J., Abis G., Gellerstedt M. (2014). Patient-reported genitourinary dysfunction after laparoscopic and open rectal cancer surgery in a randomized trial (COLOR II). Br. J. Surg.

[54] Kim H.J., Choi G.S., Park J.S., Park S.Y., Yang C.S., Lee H.J (2018). The impact of robotic surgery on quality of life, urinary and sexual function following total mesorectal excision for rectal cancer: a propensity score-matched analysis with laparoscopic surgery. Colorectal Dis.

[55] Lee S. H., Lim S., Kim J. H., Lee K. Y. (2015). Robotic versus conventional laparoscopic surgery for rectal cancer: systematic review and meta-analysis. Ann. Surg. Treat. Res.

[56] Pontallier A., Denost Q., Van Geluwe B., Adam J.P., Celerier B., Rullier E. (2016). Potential sexual function improvement by using transanal mesorectal approach for laparoscopic low rectal cancer excision. Surg. Endosc.

[57] Kneist W., Wachter N., Paschold M., Kauff D.W., Rink A.D., Lang H. (2016). Midterm functional results of taTME with neuromapping for low rectal cancer. Tech. Coloproctol.

[58] Koedam T.W.A., Veltcamp Helbach M., Van de Ven P.M. (2018). Transanal total mesorectal excision for rectal cancer: evaluation of the learning curve. Tech. Coloproctol.

[59] Taylor F.G., Quirke P., Heald R.J. (2014). Preoperative magnetic resonance imaging assessment of circumferential resection margin predicts disease-free survival and local recurrence: 5-Year follow-up results of the MERCURY Study. J. Clin. Oncol.

[60] Battersby N.J., How P., Moran B., Stelzner S. (2016). Prospective Validation of a Low Rectal Cancer Magnetic Resonance Imaging Staging System and Development of a Local Recurrence Risk Stratification Model. Ann. Surg.

[61] Baik S.H., Kim N.K., Lee K.Y. (2008). Factors Influencing Pathologic Results after Total Mesorectal Excision for Rectal Cancer: Analysis of Consecutive 100 Cases. Ann. Surg. Oncol.

